# Prediction of Glucose Tolerance without an Oral Glucose Tolerance Test

**DOI:** 10.3389/fendo.2018.00082

**Published:** 2018-03-19

**Authors:** Rohit Babbar, Martin Heni, Andreas Peter, Martin Hrabě de Angelis, Hans-Ulrich Häring, Andreas Fritsche, Hubert Preissl, Bernhard Schölkopf, Róbert Wagner

**Affiliations:** ^1^Department of Empirical Inference, Max Planck Institute for Intelligent Systems, Tübingen, Germany; ^2^Aalto University, Helsinki, Finland; ^3^Department of Internal Medicine IV, Division of Endocrinology, Diabetology, Nephrology, Vascular Disease and Clinical Chemistry, University Hospital of Tübingen, Tübingen, Germany; ^4^Institute for Diabetes Research and Metabolic Diseases of the Helmholtz Centre Munich at the University of Tübingen (IDM), Tübingen, Germany; ^5^German Center for Diabetes Research (DZD), Neuherberg, Germany; ^6^Institute of Pharmaceutical Sciences, Interfaculty Centre for Pharmacogenomics and Pharma Research, Department of Pharmacy and Biochemistry, University of Tübingen, Tübingen, Germany

**Keywords:** clinical study, supervised machine learning, oral glucose tolerance test, prediction, classification, impaired glucose tolerance, test-retest variability, machine learning classification

## Abstract

**Introduction:**

Impaired glucose tolerance (IGT) is diagnosed by a standardized oral glucose tolerance test (OGTT). However, the OGTT is laborious, and when not performed, glucose tolerance cannot be determined from fasting samples retrospectively. We tested if glucose tolerance status is reasonably predictable from a combination of demographic, anthropometric, and laboratory data assessed at one time point in a fasting state.

**Methods:**

Given a set of 22 variables selected upon clinical feasibility such as sex, age, height, weight, waist circumference, blood pressure, fasting glucose, HbA1c, hemoglobin, mean corpuscular volume, serum potassium, fasting levels of insulin, C-peptide, triglyceride, non-esterified fatty acids (NEFA), proinsulin, prolactin, cholesterol, low-density lipoprotein, HDL, uric acid, liver transaminases, and ferritin, we used supervised machine learning to estimate glucose tolerance status in 2,337 participants of the TUEF study who were recruited before 2012. We tested the performance of 10 different machine learning classifiers on data from 929 participants in the test set who were recruited after 2012. In addition, reproducibility of IGT was analyzed in 78 participants who had 2 repeated OGTTs within 1 year.

**Results:**

The most accurate prediction of IGT was reached with the recursive partitioning method (accuracy = 0.78). For all classifiers, mean accuracy was 0.73 ± 0.04. The most important model variable was fasting glucose in all models. Using mean variable importance across all models, fasting glucose was followed by NEFA, triglycerides, HbA1c, and C-peptide. The accuracy of predicting IGT from a previous OGTT was 0.77.

**Conclusion:**

Machine learning methods yield moderate accuracy in predicting glucose tolerance from a wide set of clinical and laboratory variables. A substitution of OGTT does not currently seem to be feasible. An important constraint could be the limited reproducibility of glucose tolerance status during a subsequent OGTT.

## Introduction

Impaired glucose tolerance (IGT) defines an intermediate state of blood glucose regulation that is not yet clearly pathologic. However, it is important to recognize this state because individuals with IGT have significantly increased incidence of type 2 diabetes (1), and it is associated with an elevated cardiovascular disease risk (2–4). Therefore, identification of IGT is important to allow focused diabetes and cardiovascular disease prevention strategies on those who are at the highest risk.

There is a widely accepted consensus definition of IGT using a cutoff of 7.8 mmol l^−1^ for the postchallenge plasma glucose level measured 2 h after the administration of a 75 g glucose load in an oral glucose tolerance test (OGTT) (5). While the test itself is usually well tolerable and not too difficult to perform, it requires considerable attention and time from both the participant and the medical personnel. Unfortunately, currently, there is no procedure of substituting the OGTT to establish IGT. For the diagnosis of diabetes, the use of glycated hemoglobin (HbA1c) levels has been proposed as an alternative to OGTT. However, it has been shown that using only HbA1c to diagnose diabetes misses more than half of the diabetes cases established by OGTT (6). According to recommendations of the American Diabetes Association, prediabetes can also be diagnosed with an intermediary HbA1c range of 5.7–6.4% (7). Nevertheless, it has recently been demonstrated that in obese individuals, 44% of prediabetes cases captured by OGTT were missed using the HbA1c criterion (8). Furthermore, the HbA1c-based diagnosis of prediabetes precludes the differentiation of impaired fasting glycemia from IGT.

Therefore, we investigated if machine learning could be utilized to differentiate IGT from normal glucose tolerance (NGT) using a battery of potential predictor variables that can be easily obtained in a fasting state. Machine learning can be employed as a computational technique to recognize specific patterns that are characteristic for a class of entities. Specifically, we set out to test supervised machine learning to predict IGT from anthropometric, clinical, and laboratory variables obtained at one time point.

## Materials and Methods

### Participants

Data of the Tuebingen Family Study (TUEF) were retrospectively analyzed. In the TUEF study, participants at an increased risk for type 2 diabetes were recruited if they had a family history of diabetes, obesity, or previously known prediabetes. All participants underwent OGTT with an extensive phenotyping including the laboratory measurements of several glycemic traits. The data were acquired between December 1996 and November 2016. To separate training and test sets, the data were longitudinally split at the date January 1, 2012. The training set comprised OGTTs performed before this date, and the test set comprised OGTTs performed after this date. To measure the intraindividual variability of an OGTT in our study center, we identified 78 participants who underwent a second OGTT within 1 year and had a weight difference <3 kg between the two measurements.

This study was carried out in accordance with the recommendations of the Ethics Committee of the University of Tübingen with written informed consent from all subjects. All subjects gave written informed consent in accordance with the Declaration of Helsinki. The protocol was approved by the Ethics Committee of the University of Tübingen.

### Model Variables

We preselected routinely phenotyped variables upon scientific credibility and feasibility as model features for machine learning. The list of selected variables with available evidence for an association with IGT or glycemia is shown in Table 1. Mean arterial blood pressure, alanine aminotransferase, family history of diabetes (9), and prolactin (10) were initially selected and then excluded due to a high missing rate (>10%). The feature variables sex, age, height, weight, fasting glucose, and the outcome variable IGT had no missing values in the training set, and all other features had missing value rates <10%. Missing values were imputed using multivariate imputation by chained equations (11). For constructing the classifiers, all variables were normalized to a mean of 0 with a SD of 1 (scaled and centered). No further preprocessing was applied.

**Table 1:** List of anthropometric, clinical, and laboratory variables used as features in the machine learning classifiers.

Variable^a^	Evidence of association with impaired glucose tolerance (IGT)
Sex	Women have higher risk of IGT (12)

Age	IGT incidence increases with age (13)

Height	Used separately as underlying variables of body mass index that is strongly associated with IGT age (13)
Weight	

Glucose	By definition strongly associated with glycemia, prediabetes, and diabetes

HBA1C	Strongly associated with IGT (14)

Hemoglobin	Potentially interacts with the association of HbA1c with the outcome (BZ120)

Mean corpuscular volume (MCV)	MCV could interact with HbA1c in modulating its association with glycemia

Ferritin	Elevated ferritin is associated with impaired glucose tolerance (15), ferritin levels may also interact with HbA1c in modulating its association with glycemia

Potassium	Associated with prediabetes in hypertensive persons (16)

Insulin	Higher fasting insulin is associated with insulin resistance and IGT (17)

C-peptide	Similar to insulin, higher levels are associated with IGT (18)

Proinsulin	A read-out of proinsulin-insulin conversion, higher levels are associated with IGT (19, 20)

Non-esterified fatty acids (NEFAS)	High-fasting free fatty acids (=NEFAS) predict diabetes (21)

Triglycerides	Well-known association with type 2 diabetes and prediabetes

Total cholesterol	Well-known association with type 2 diabetes and prediabetes

LDL cholesterol	Well-known association with type 2 diabetes and prediabetes

HDL cholesterol	Well-known inverse association with type 2 diabetes and prediabetes

C-reactive protein (CRP)	Elevated CRP predicts the development of type 2 diabetes (22)

Aspartate aminotransferase (AST)	AST is associated with fatty liver and IGT (23)

Gamma-glutamyl transferase (GGT)	GGT is associated with IGT (24)

Uric acid	Associated with IGT especially in women (25)

*^a^Clinical chemistry values have been determined from fasting plasma*.

### OGTT and Laboratory Measurements

All participants received a 75-g glucose solution (Accu-Check Dextro, Roche) at 8:00 a.m. following an overnight fast. Venous blood was obtained through an indwelling venous catheter before and 30, 60, 90, and 120 min after glucose ingestion. Glucose values were measured directly using a bedside glucose analyzer (YSI, Yellow Springs, CO, USA). All other obtained blood samples were put on ice, and the serum was centrifuged within 2 h. Plasma insulin and C-peptide were determined by an immunoassay with the ADVIA Centaur XP Immunoassay System (Siemens Healthineers, Eschborn, Germany).

Serum proinsulin concentrations were measured using a microparticle enzyme immunoassay (IBL, Hamburg, Germany) on a BEP III System (Siemens Healthineers, Eschborn, Germany). Triglycerides (TGs) and total, HDL, and low-density lipoprotein cholesterol levels, as well as alanine aminotransferase, aspartate aminotransferase, and gamma-glutamyl transferase (GGT) activities, were measured using the ADVIA XPT clinical chemical analyzer (Siemens Healthineers, Eschborn, Germany). Plasma concentrations of total non-esterified fatty acid (NEFA) were measured with an enzymatic method (WAKO Chemicals, Neuss, Germany) on the latter instrument. Hematological parameters, including mean corpuscular volume, were determined on the Sysmex XN-10 (Sysmex GmbH, Norderstedt, Germany) or ADVIA 2120 hematological analyzers (Siemens Healthineers, Eschborn, Germany). HbA1c measurements were performed using the Tosoh glycohemoglobin analyzer HLC-723G8 (Tosoh Bioscience Tokyo Japan).

### Model Computation and Statistics

All computations were run under R version 3.4 (26). Classifiers were computed using the wrapper package Classification and Regression Training (27). Detailed information on the machine learning packages is provided as Supplementary Material; see Table S1 in Supplementary Material. Model optimization in the training set was performed by fivefold cross-validation, with three sets of repeats. The granularity of the tuning parameter grid (“tuneLength”) was set to 5 (default: 3). We used Synthetic Minority Over-sampling Technique to compensate for the imbalanced prevalence of IGT and NGT in the training set (28). By doing this, we simulate balanced NGT and IGT prevalence for the classifier, thus precluding it from utilizing prevalence information in the estimation procedure. Intraindividual percentage error was calculated as the ratio of the difference of two measurements divided by their mean. Intraindividual coefficient of variation was calculated as the ratio of SD and mean. Insulin sensitivity was assessed using the method of Matsuda and DeFronzo (29).

## Results

### Machine Learning Classifiers

We tested the performance of 10 machine learning classifiers to predict IGT from 22 biologically reasonable feature variables. Table 2 compares the characteristics of the training and test sets. In the training set during resampling by repeated cross-validation, the highest model accuracy indicating the proportion of right predictions over all predictions was shown for the recursive partitioning and regression trees (RPART) classifier at a median of 0.82 (interquartile range, 0.80–0.83). In the independent test set, the same RPART classifier reached the highest accuracy of 0.78. The mean sensitivity across all models was 0.67 ± 0.08, and the specificity was 0.75 ± 0.08. The generalized linear model and the penalized multinomial regression classifier had the highest sensitivity (both 0.74), while the highest specificity (0.88) was yielded by the RPART classifier. These measures translate to positive predictive values of around 51 ± 6% and negative predictive values of 86 ± 2%. Model accuracy showed a mean of 0.73 ± 0.04.

**Table 2:** Characteristics of the training and test set for the feature variables and the target variable defining the classification.

	Training set			Test set			
	*N*	Mean	SD	*N*	Mean	SD	p*
Sex (f/m)	2,337			929			0.79
Age (years)	2,337	40	13	929	49	15	<0.0001
Height (cm)	2,337	171	9	929	170	9	0.00022
Weight (kg)	2,337	91.5	29.1	929	89.8	25.5	0.11
Fasting glucose (mmol l^−1^)	2,337	5.25	0.72	929	5.46	0.74	<0.0001
Glycated hemoglobin HbA1c (%)	2,190	5.4	0.5	916	5.7	0.5	<0.0001
Hemoglobin (g dl^−1^)	2,208	13.8	1.2	917	13.9	1.2	0.047
Mean corpuscular volume (fl)	2,208	86	5	917	86	4	0.59
Potassium (mmol l^−1^)	2,155	3.97	0.32	912	3.99	0.35	0.13
Fasting insulin	2,304	84	75	919	110	77	<0.0001
C-peptide (pmol l^−1^)	2,236	685	344	913	603	308	<0.0001
Triglycerides (mg dl^−1^)	2,185	132	152	917	123	71.9	0.027
Cholesterol (mg dl^−1^)	2,183	194	38.9	917	197	40.1	0.052
Low-density lipoprotein (mg dl^−1^)	2,157	121	33.5	917	115	34.1	<0.0001
HDL (mg dl^−1^)	2,157	53.3	14.3	917	53.4	13.9	0.99
Uric acid (mg dl^−1^)	2,174	5.5	1.4	836	5.6	1.3	0.16
Aspartate aminotransferase (U l^−1^)	2,132	22	11	917	24	10	<0.0001
Gamma-glutamyl transferase (U l^−1^)	2,167	28	33	917	28	36	0.71
C-reactive protein (mg dl^−1^)	2,161	0.42	0.62	917	0.36	0.51	0.0038
Ferritin (μg dl^−1^)	2,175	9	12	834	12	14	<0.0001
Non-esterified fatty acids (μmol l^−1^)	2,210	593	251	906	606	225	0.15
Proinsulin (pmol l^−1^)	2,132	6	6.5	890	3.6	3.9	<0.0001
Postchallenge glucose (mmol l^−1^)	2,337	6.65	2.15	929	6.96	2.19	0.00022

*t-Test or Fisher’s exact test, as appropriate.

Simple accuracy measurements could be biased because the predicted categories were unbalanced. IGT has a prevalence of 27%, corresponding to an NGT prevalence of 73% in the test set, such that a uniform “forecast” of NGT would also result in an accuracy of 0.73. This value is also called no information rate. To quantify prediction accuracy adjusted for the expected accuracy, Cohen’s kappa (κ) was applied as a suitable measure. Table 3 shows the accuracy, κ statistic, and the *p*-value of the difference of κ from the no information rate for all models in the test set. The mean κ over all models in the test set was 0.38 ± 0.04.

**Table 3:** Model performance showing crude accuracy values (the ratio of right predictions over all predictions) and κ statistic (accuracy in relation to expected accuracy) for the evaluated machine learning classifiers in the test set.

Method	Accuracy	κ	*p* Value
Recursive partitioning (rpart)	0.783	0.423	<0.0001
Lasso (glmnet)	0.767	0.418	0.003
Stochastic gradient boosting (gbm)	0.761	0.414	0.012
Random forest (rf)	0.744	0.412	0.142
Extended gradient boost (xgbLinear)	0.74	0.394	0.22
generalized additive model (gamLoess)	0.708	0.368	0.913
Neural networks (nnet)	0.695	0.348	0.987
Generalized linear model (glm)	0.686	0.339	0.998
Penalized multinomial regression (multinom)	0.686	0.339	0.998
Partial least squares (pls)	0.692	0.331	0.993

*A one-sided binomial test *p* value is shown for the difference of the model accuracy from the no information rate. The no information rate is equivalent to the prevalence of impaired glucose tolerance in the test set (0.73)*.

**Figure 1:**
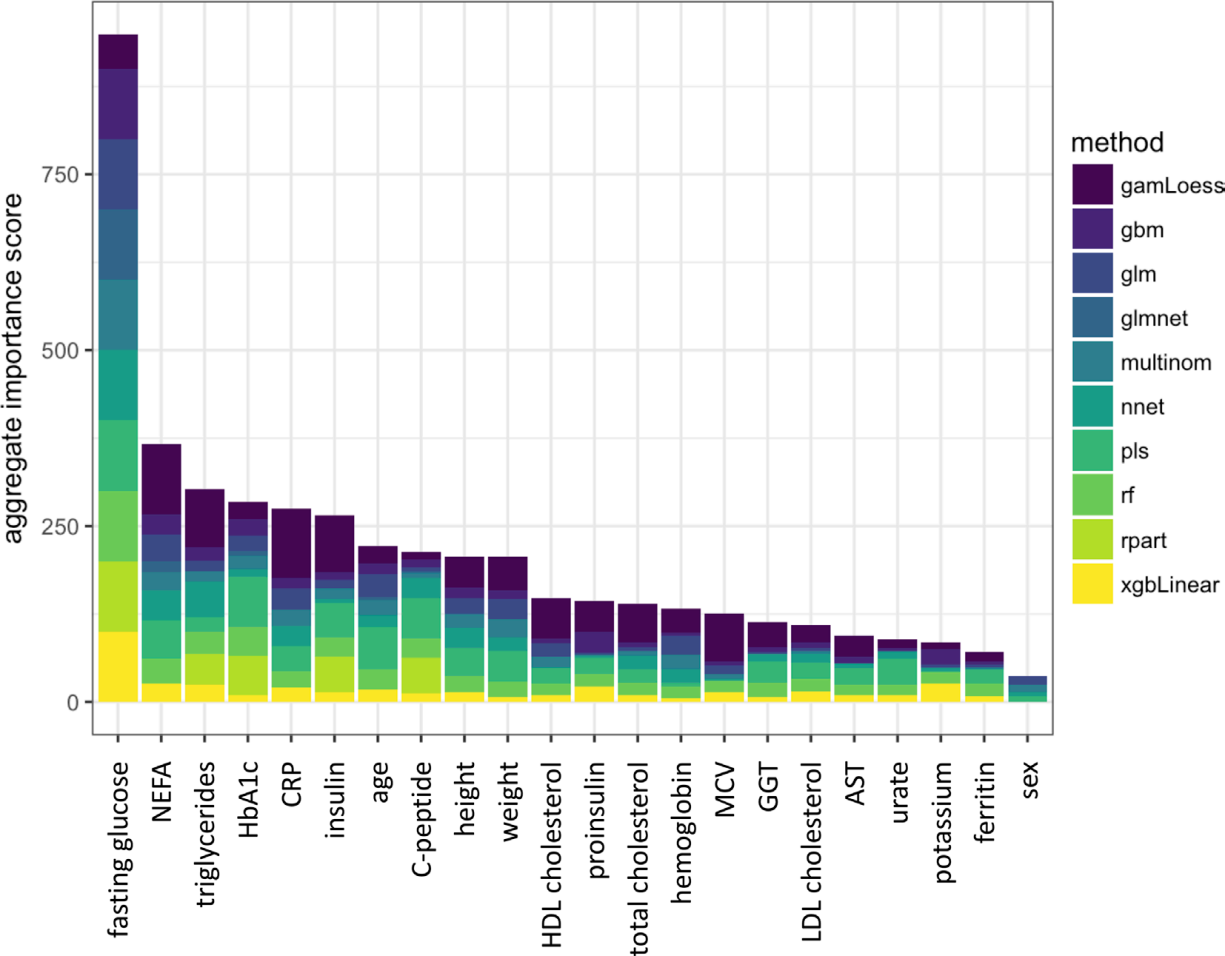
Aggregated importance score in the machine learning classifiers for each feature variable. Individual importance scores are represented by colors in the stacked bars. The classifiers are described in Table S1 in Supplementary Material.

Furthermore, to show which predictor variables are used by the machine learning classifiers, model-specific variable importance measures were obtained for each model. By using a common scale of 0–100 (with 0 indicating an omitted variable and 100 indicating the variable with the highest importance), we show overall variable importance in Figure 1. The single most important model feature was fasting glucose. It is followed by NEFA, TGs, and HbA1c. Some of the tested machine learning methods shrink the number of model features from the original 22 by completely eliminating weak predictor variables. For example, the best-performing RPART classifier has only retained fasting glucose, HbA1c, insulin, C-peptide, and TGs, while the lasso method used fasting glucose, NEFA, C-peptide, HbA1c, age, height, and CRP for the classifier.

To compare model performance in insulin-sensitive and insulin-resistant individuals, we tested the classifiers in subsets of the test set split at the median insulin sensitivity index. Higher accuracy levels were obtained in the insulin-sensitive subset; however, κ was generally very low, probably due to the low proportion of individuals with IGT among insulin-sensitive subjects. In contrast, accuracy and κ values were moderate in individuals with low insulin sensitivity.

### Intraindividual Variability of OGTT

To compare the predictive performance of machine learning models with the predictive performance of an earlier OGTT for discriminating IGT from NGT in an individual, we investigated 78 participants who received a second OGTT within 1 year of their first OGTT. Only participants who did not undergo lifestyle intervention in between and who did not experience substantial weight change (<3 kg) were selected. Postchallenge 120-min glucose in a repeated OGTT, i.e., the underlying variable for discrimination of IGT from NGT, showed large variation. The percentage error of measurement pairs for postchallenge 120-min glucose, calculated as the average of the bias-to-mean ratio of the individual data points of the plot, was 18.3% (±15.2%). For comparison, fasting glucose had a percentage error of only 6.4% (±5.0%). Bland-Altman plots and coefficients of variation of repeated OGTT measurements for each time point are shown in the Supplementary Material, Figures S1 and S2 in Supplementary Material, respectively. The calculated mean coefficients of variation were 13.0% (±11.0%) for postchallenge glucose and 4.6% (±3.5%) for fasting glucose. By using these data, we calculated the predictive accuracy of one OGTT for forecasting IGT in a second OGTT. In the set of 78 OGTT measurement pairs, the agreement (accuracy) between corresponding measurements was 0.77. Given the expected frequency of IGT, the computed κ statistic was 0.46.

## Discussion

Our work shows that machine learning is capable of predicting the glucose tolerance status by 22 baseline variables obtained at fasting blood acquisition. The best-performing RPART classifier had an unbiased predictive accuracy κ of 0.42, which is a moderate classifier according to the consideration of Landis and Koch (30). From another aspect, given a test population with similarly high IGT prevalence, this classifier’s IGT prediction would be correct in 62% of the cases (positive predictive value), and the NGT predictions would be correct in 83% of the cases (negative predictive value). This might fall short of initial expectations. However, the κ value of the RPART classifier (0.42) approaches the computed intraindividual κ of a repeated OGTT (0.46) that can be interpreted as the upper bound of a feasible prediction of IGT. A wealth of studies has been investigating the reproducibility of OGTT results since the 1960s (31–36). It has been shown that in a population with NGT, the 95th percentile of random test–retest differences is 46% for the postchallenge glucose, while this ratio is only 16% for fasting glucose values (36). The coefficient of variation has been estimated around 16–17% for 2-h postchallenge glucose levels (34, 36). In our population, the coefficient of variation was only 12%, which might be due to the stringent laboratory methods employed in our single-center study. Higher variability seems to be also true for other postchallenge analytes such as insulin levels during the OGTT (32). Fasting levels of these analytes are more stable, but still prone to a relatively high intraindividual variance. For example, the intraindividual coefficient of variation for fasting TGs is 25–35% (37, 38). Since these analytes are feature variables in the machine learning classifiers, they introduce a further noise to the prediction.

As the aggregate statistics of variable importance shows, from the 22 initially selected feature variables, the top 5 variables were fasting glucose, NEFA, TGs, HbA1c, C-peptide, and CRP. One could speculate that fasting glucose, TGs, and NEFA might reflect insulin resistance, while lower C-peptide levels associate with a dysfunction of insulin secretion. HbA1c directly correlates with glycemia, such that higher postprandial glucose levels that are present with IGT contribute to an elevation of HbA1c. Interestingly, in one of our recent works, NEFA emerged as a very robust proxy for the estimation of insulin resistance (39). The emergence of CRP among the top feature variables underlines the role of subclinical inflammation in the pathogenesis of IGT and prediabetes (40). In the aggregated variable, importance score fasting insulin is unexpectedly only the sixth most important variable closely following CRP. However, the best-performing RPART classifier just retained fasting glucose, HbA1c, insulin, C-peptide, and TGs. This and the relatively small difference among the aggregate importance scores of these variables suggest that some of the most important feature variables at position 2–6 of the aggregate variable importance list relate to similar biological aspects and can be used interchangeably. This might be true for TGs and NEFA as well as, to some extent, C-peptide and insulin levels.

The most important limitation of our work is that we cannot generalize the classifier on different study populations and different study settings. In our subset analyzes, the classifiers seemed to yield a higher accuracy in the insulin sensitive, and a lower accuracy in the insulin-resistant subgroup. Kappa was strongly influenced by the different IGT rates in the two subsets and was generally lower than in the original test set. The retraining of classifiers could improve model performance in study populations with substantially different insulin sensitivity distributions. A further limitation of our study is the large set of clinical and laboratory variables, some of which, e.g., NEFA or proinsulin, will not be easily accessible in every laboratory. Also, a precise determination of NEFA levels requires careful preanalytical handling.

Taken together, prediction of IGT from baseline variables with supervised machine learning is a feasible technique. However, in spite of the complex analytes as feature variables, predictive accuracy remains moderate. Therefore, stringently performed OGTT still remains the gold standard for determining IGT. Prediction of IGT with machine learning could be employed to fill in IGT status when OGTT is technically not possible or to retroactively estimate IGT status from stored fasting samples.

## Ethics Statement

This study was carried out in accordance with the recommendations of the Ethics Committee of the University of Tübingen with written informed consent from all subjects. All subjects gave written informed consent in accordance with the Declaration of Helsinki. The protocol was approved by the Ethics Committee of the University of Tübingen.

## Author Contributions

RB and RW analyzed the data and wrote the manuscript. MH, AP, AF, and HP contributed to the interpretation of data and edited the manuscript. MH, H-UH, and BS contributed to the study design and interpretation of data and reviewed the manuscript.

## Conflict of Interest Statement

The authors declare that the research was conducted in the absence of any commercial or financial relationships that could be construed as a potential conflict of interest.
