# Supplementary material for: Prediction of Glucose Tolerance without an Oral Glucose Tolerance Test
Source: Front Endocrinol (Lausanne). 2018 Mar 19;9:82. doi: 10.3389/fendo.2018.00082 (PMC5868129; doi:10.3389/fendo.2018.00082)

Supplementary Figures

Supplementary Figure 1

Difference and mean of pairwise glucose measurements from intra-individual OGTTs at every assessed OGTT-timepoint. Red line indicates zero difference (identic values at repeated measurements), dashed blue lines indicate means of all positive and negative deviations.

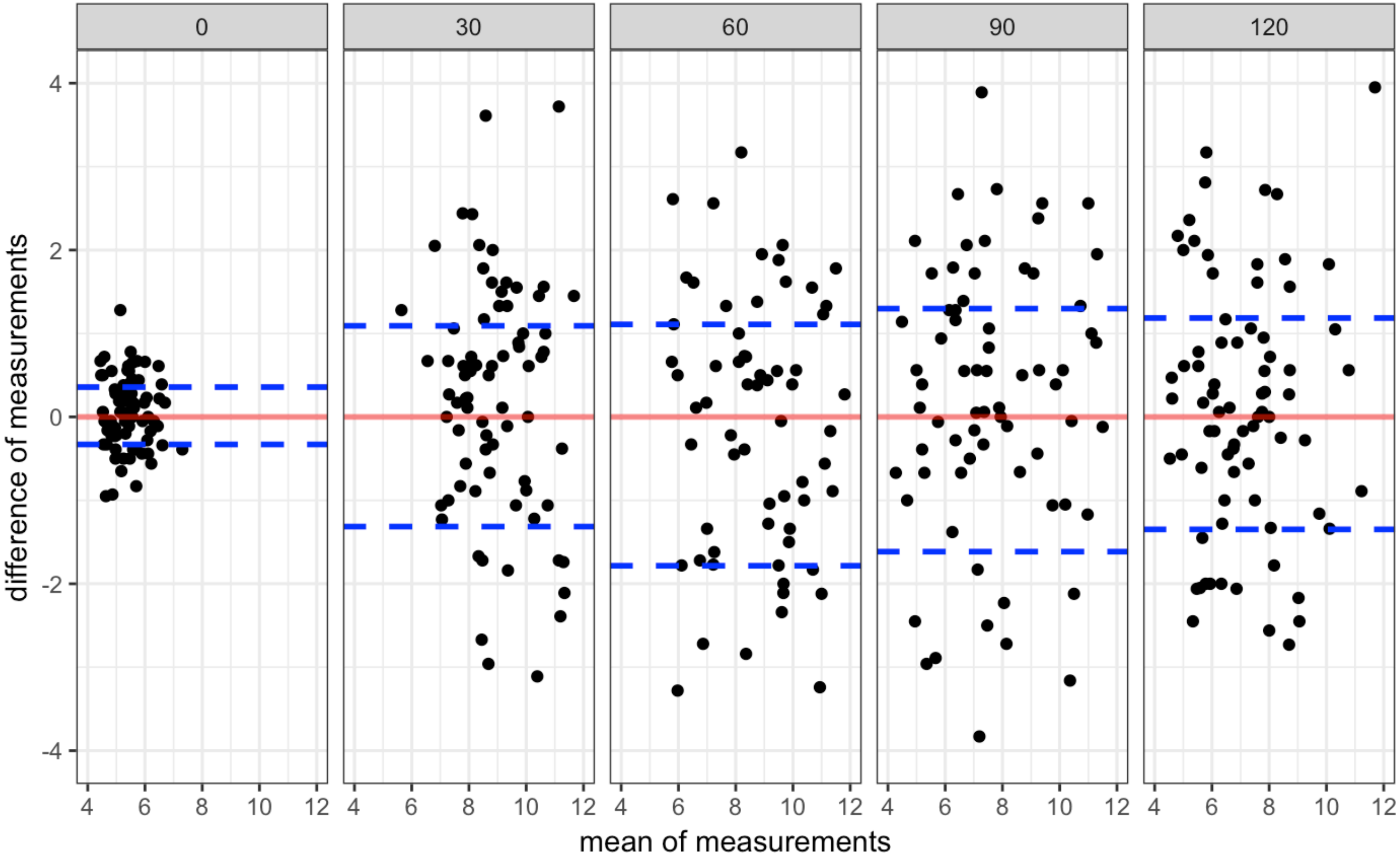

Supplement: Supplementary file 1 [file image_1.PDF]
