# Supplementary material for: Prediction of Glucose Tolerance without an Oral Glucose Tolerance Test
Source: Front Endocrinol (Lausanne). 2018 Mar 19;9:82. doi: 10.3389/fendo.2018.00082 (PMC5868129; doi:10.3389/fendo.2018.00082)

Supplementary Figure 2

Between measurement percent error (difference/mean) of intra-individual glucose measurements plotted against the means of the glucose value pairs at every assessed OGTT-timepoint

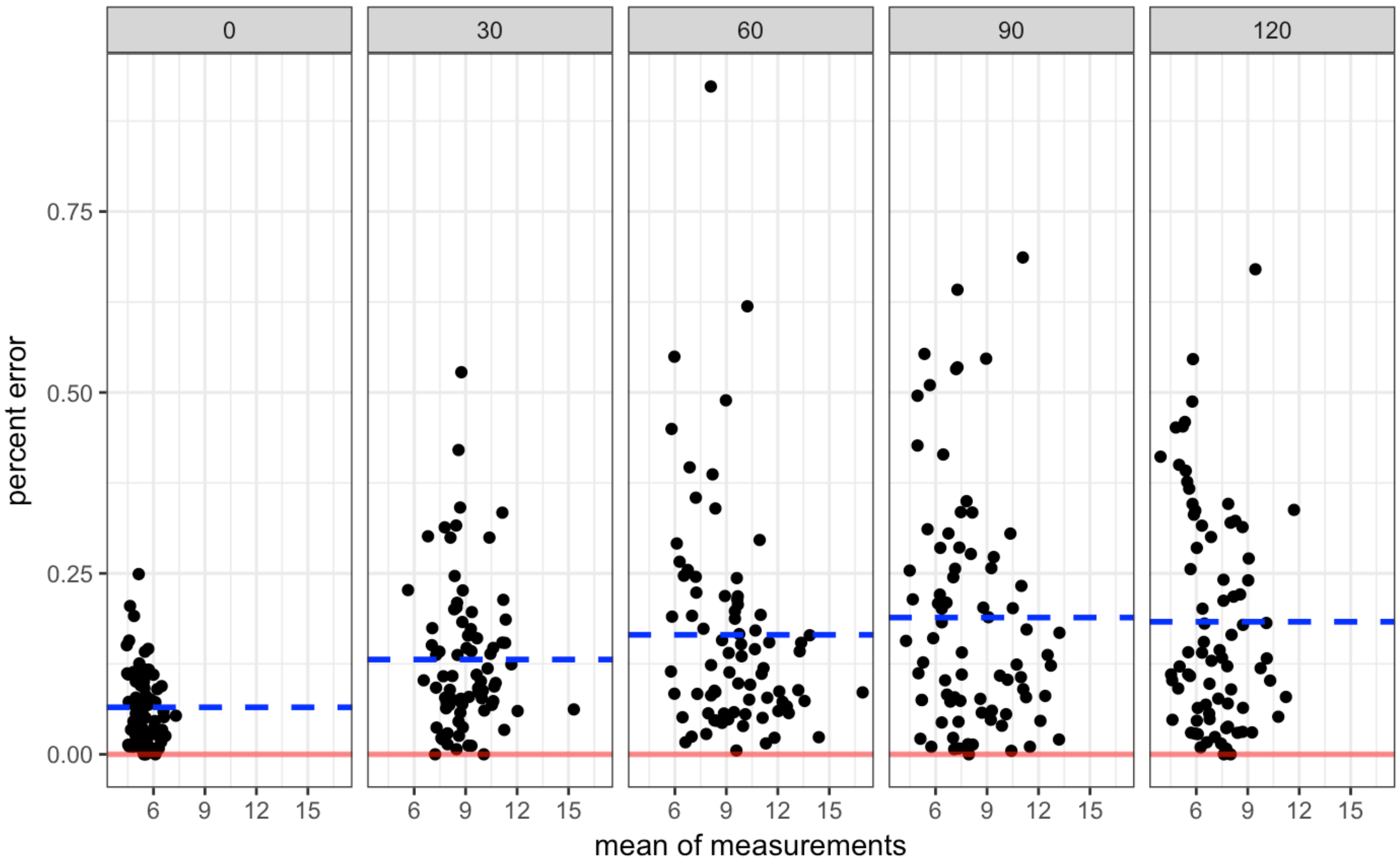

Supplement: Supplementary file 2 [file image_2.PDF]
