# Supplementary material for: Prediction of Glucose Tolerance without an Oral Glucose Tolerance Test
Source: Front Endocrinol (Lausanne). 2018 Mar 19;9:82. doi: 10.3389/fendo.2018.00082 (PMC5868129; doi:10.3389/fendo.2018.00082)
Supplement: Supplementary file 3 [file table_1.PDF]

Supplementary Material

Supplementary Table 1

Reference to the used machine learning classifiers

**method**	**description**	**R package**	**tuning parameters varying over the grid search**
gbm	stochastic gradient boosting	gbm	interaction depth, n.trees
rpart	recursive partitioning	CART	cp
xgbLinear	extended gradient boost	xgboost	lambda, alpha, nrounds
rf	random forest	rf	mtry
glmnet	lasso	glmnet	alpha, lambda
glm	generalized linear model	glm	
gamLoess	generalized additive model, local ("loess") regression	gam	
pls	partial least squares	pls	ncomp
multinom	penalized multinomial regression	nnet	decay
nnet	neural networks	nnet	
