# Supplementary material for: Prediction of Glucose Tolerance without an Oral Glucose Tolerance Test
Source: Front Endocrinol (Lausanne). 2018 Mar 19;9:82. doi: 10.3389/fendo.2018.00082 (PMC5868129; doi:10.3389/fendo.2018.00082)
Supplement: Supplementary file 4 [file table_2.PDF]

Supplementary Table 2

Machine learning classifiers tested in subsets of the test data with different insulin sensitivity (ISI). Insulin sensitivity was assessed with the Matsuda index, data were split by the median. In the low ISI group,

	test set		subset with low ISI		subset with high ISI	
	27% IGT		44% IGT		11% IGT	
Classifier	Accuracy	Kappa	Accuracy	Kappa	Accuracy	Kappa
rpart	0.783	0.423	0.69	0.359	0.865	0.209
glmnet	0.767	0.418	0.659	0.308	0.865	0.295
gbm	0.761	0.414	0.665	0.33	0.854	0.201
rf	0.744	0.412	0.639	0.292	0.84	0.256
xgbLinear	0.74	0.394	0.639	0.29	0.836	0.196
gamLoess	0.708	0.368	0.612	0.249	0.798	0.239
nnet	0.695	0.348	0.603	0.236	0.778	0.17
glm	0.686	0.339	0.592	0.217	0.772	0.202
multinom	0.686	0.339	0.592	0.217	0.772	0.202
pls	0.692	0.331	0.596	0.218	0.78	0.15
